# Human Induced Pluripotent Stem Cell-Derived Astrocytes Are Differentially Activated by Multiple Sclerosis-Associated Cytokines

**DOI:** 10.1016/j.stemcr.2018.09.015

**Published:** 2018-10-25

**Authors:** Sylvain Perriot, Amandine Mathias, Guillaume Perriard, Mathieu Canales, Nils Jonkmans, Nicolas Merienne, Cécile Meunier, Lina El Kassar, Anselme L. Perrier, David-Axel Laplaud, Myriam Schluep, Nicole Déglon, Renaud Du Pasquier

**Affiliations:** 1 Laboratory of Neuroimmunology, Neuroscience Research Centre, Department of Clinical Neurosciences, CHUV, Lausanne, Switzerland; 2 Laboratory of Neurotherapies and NeuroModulation, Neuroscience Research Centre, Department of Clinical Neurosciences, CHUV, Lausanne, Switzerland; 3 Institute for Stem Cell Therapy and Exploration of Monogenic Diseases (I-Stem), Corbeil-Essonnes, France; 4 Institut National de la Santé et de la Recherche Médicale (INSERM) UMR861, I-Stem, AFM, Corbeil-Essonnes, France; 5 Centre de Recherche en Transplantation et Immunologie UMR1064, INSERM, Université de Nantes, Nantes, France; 6 Service of Neurology, Department of Clinical Neurosciences, CHUV, CHUV BH-10/131, 46, rue du Bugnon, Lausanne 1011, Switzerland

**Keywords:** induced pluripotent stem cells, differentiation, astrocytes, multiple sclerosis, neuroinflammation

## Abstract

Recent studies highlighted the importance of astrocytes in neuroinflammatory diseases, interacting closely with other CNS cells but also with the immune system. However, due to the difficulty in obtaining human astrocytes, their role in these pathologies is still poorly characterized. Here, we develop a serum-free protocol to differentiate human induced pluripotent stem cells (hiPSCs) into astrocytes. Gene expression and functional assays show that our protocol consistently yields a highly enriched population of resting mature astrocytes across the 13 hiPSC lines differentiated. Using this model, we first highlight the importance of serum-free media for astrocyte culture to generate resting astrocytes. Second, we assess the astrocytic response to IL-1β, TNF-α, and IL-6, all cytokines important in neuroinflammation, such as multiple sclerosis. Our study reveals very specific profiles of reactive astrocytes depending on the triggering stimulus. This model provides ideal conditions for in-depth and unbiased characterization of astrocyte reactivity in neuroinflammatory conditions.

## Highlights

• Human iPSCs are differentiated from blood of control and multiple sclerosis subjects
• Functional astrocytes are differentiated from hiPSC in serum-free conditions
• In-depth characterization of the effect of serum on astrocyte phenotype
• Multiple sclerosis cytokines trigger specific activation profiles in astrocytes

## Graphical Abstract

**Figure:**
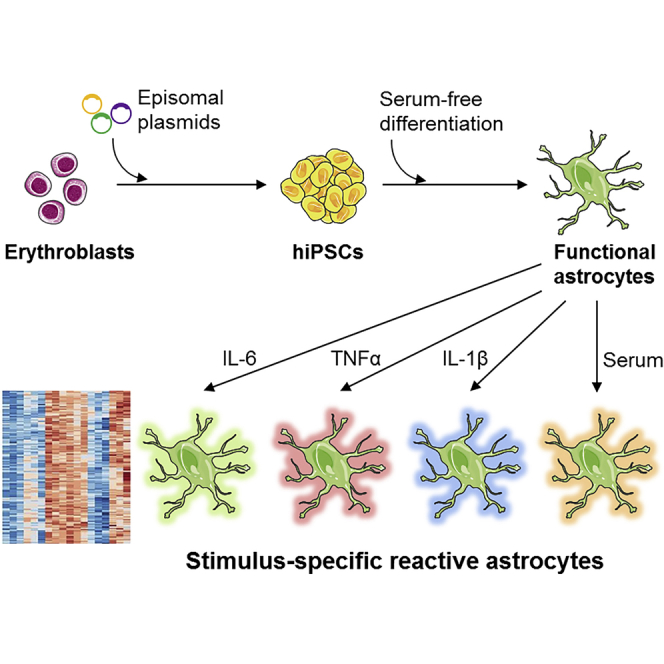

## Teaser

Perriot et al. describe a new protocol to differentiate both resting and reactive astrocytes from human induced pluripotent stem cells in serum-free conditions. They show that astrocytes exhibit highly contrasted responses to serum and to cytokines involved in multiple sclerosis, establishing that this model is suitable to study neuroinflammation.

## Introduction

Astrocytes represent a very large proportion of CNS cells and exercise major roles in CNS development and adult life. In healthy conditions, their primary task is to provide physical and trophic support to other CNS cells, to ensure proper synaptic maturation and signaling (Nedergaard et al., 2003), as well as assuring blood-brain barrier integrity (Sofroniew and Vinters, 2010). In addition to these physiological functions, astrocytes display high sensitivity to modifications of their environment, undergoing diverse changes in morphology, gene expression, and functionalities (Khakh and Sofroniew, 2015). Astrocytes are fully integrated in the immune response of the CNS and participate in the first line of defense against infection (Farina et al., 2007). But, astrocytes can also be noxious: they seem to actively contribute to the pathogenesis of several neurodegenerative diseases, such as Alzheimer's disease, Parkinson's disease, or amyotrophic lateral sclerosis (Capani et al., 2016). Using two different models of CNS injury, recent studies have shown that neuroinflammation and ischemia trigger two different profiles of reactive astrocytes termed A1 and A2, the first one being associated with a harmful phenotype and the latter a protective one (Liddelow et al., 2017, Zamanian et al., 2012). However, other *in vivo* models revealed a still more complex role of astrocytes, which can be protective during the early phases of neuroinflammation (Colombo and Farina, 2016), but detrimental during chronic CNS inflammation (Mayo et al., 2014). These data highlight the complex regulation of astrocyte reactivity during neuroinflammation and call for a precise characterization of astrocyte-activating stimuli.

Multiple sclerosis (MS) is an example of auto-inflammatory disease of the CNS where astrocytes are likely strongly involved: this disease is characterized by demyelination followed by axonal loss and ultimately neurodegeneration. In MS, activated immune cells from the periphery migrate to the CNS where they drive injuries to the nervous tissue (Sospedra and Martin, 2005). In this pathology, reactive astrocytes are present in and near demyelinated lesions (Brosnan and Raine, 2013, Perriard et al., 2015). Interestingly, some drugs used to treat MS such as interferon-β (Rothhammer et al., 2016), fingolimod (Rothhammer et al., 2017), and dimethylfumarate (Galloway et al., 2017) have been shown to redirect reactive astrocytes toward a more protective phenotype.

Nevertheless, compared with the abundant literature available from mouse models, the number of studies assessing reactivity of human astrocytes is limited. Yet, there are significant differences between rodent and human astrocytes at basal levels (Zhang et al., 2016) and following inflammatory stimuli (Tarassishin et al., 2014). In particular, the understanding of astrocyte phenotypes in human diseases has been hampered by the very limited access to CNS samples from patients.

In this context, human induced pluripotent stem cells (hiPSCs) represent a major technological advance to study CNS disease-related mechanisms. A few groups have generated hiPSC-derived astrocytes, but these methods remain challenging, often requiring long and/or technically complicated protocols (Krencik and Zhang, 2011, Tyzack et al., 2016). Furthermore, many of the previously published studies lack data addressing reproducibility of differentiation over several hiPSC lines, or in-depth characterization of functionality and phenotype of the astrocytes generated (Chandrasekaran et al., 2016). Despite the strong implication of astrocytes in neuroinflammation, the reactivity upon stimulation of hiPSC-derived astrocytes has been addressed in few studies (Lundin et al., 2018, Roybon et al., 2013, Santos et al., 2017, Tcw et al., 2017) and all studies but one used fetal bovine serum (FBS) to differentiate astrocytes. Yet, such serum is known to induce long-term changes in inflammation-related gene expression (Zhang et al., 2016).

Here, we derived iPSCs from three healthy controls and four MS patients. We describe a method to generate mature and fully functional astrocytes from hiPSCs in serum-free media, thus resting astrocytes having the capacity to react to inflammatory stimuli. Indeed, we show that differentiating astrocytes from hiPSCs in the presence of serum has a profound impact on astrocyte phenotype and reactivity. Finally, in an effort to better characterize reactive astrocyte phenotypes in neuroinflammation, we assess distinct reactive astrocyte profiles triggered by different neuroinflammatory stimuli particularly implicated in MS.

## Results

### Mature Human Astrocytes Are Derived from Induced Pluripotent Stem Cells of Control Subjects and MS Patients in Serum-free Conditions

We generated hiPSC lines from blood of three healthy controls and four MS patients (Figure S1A). Human iPSCs were characterized to ensure a normal karyotype, pluripotency, and capacity to differentiate (Figures S1B–S1D). All hiPSC lines displayed similar pluripotency and differentiation profiles and only hiPSC lines exhibiting a normal karyotype were selected for the study. Two hiPSC lines per subject were differentiated into astrocytes (except for HC1 for which there was one hiPSC line).

**Figure 1:**
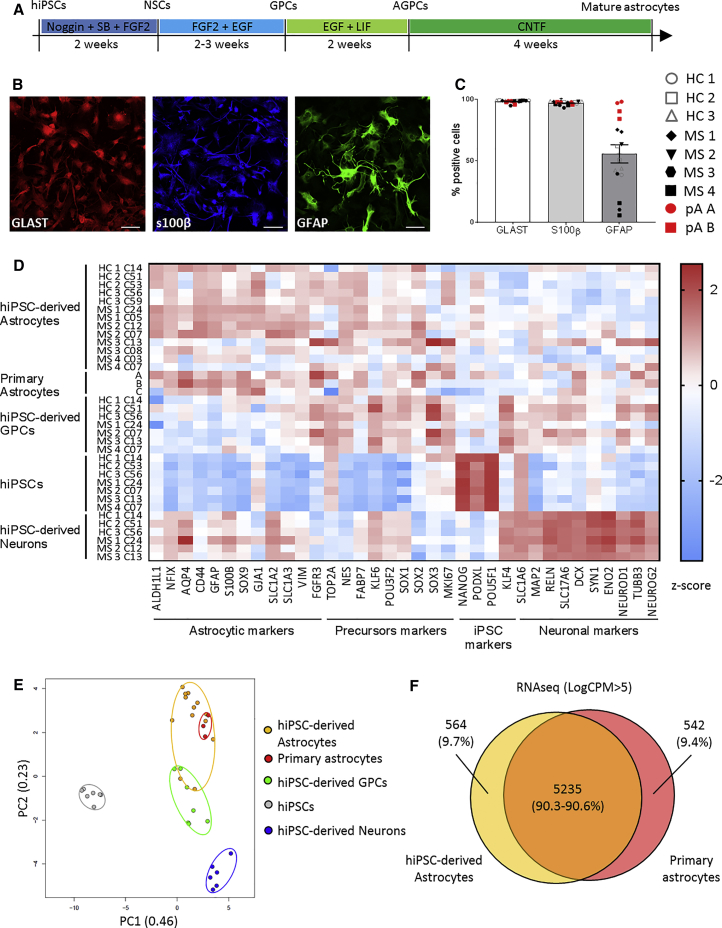
iPSCs from Control Subjects and MS Patients Efficiently Differentiate into Mature Astrocytes Astrocytes were derived from seven donors (three healthy controls [HCs] and four MS). Two hiPSC lines were derived for each study subject except for HC1 (one hiPSC line). (A) Timeline representing the different steps of hiPSC differentiation into mature astrocytes. (B) Representative immunofluorescence staining images of hiPSC-derived astrocytes expressing GLAST (red), S100β (blue), and GFAP (green) obtained from healthy control HC2. Scale bar, 100 μm. Pictures were acquired with a Zeiss LSM 880 confocal microscope. (C) Expression of the markers GLAST, S100β, and GFAP in hiPSC-derived astrocytes and primary astrocytes as assessed by flow cytometry. Bars represent the global mean ± SEM. Wilcoxon test was performed to assess statistical significance (^∗^p < 0.05). Each experiment was performed in duplicate and each dot represents the mean results of one or two experiments performed on a given cell line. (D) Gene expression of 35 markers from hiPSC-derived astrocytes, primary astrocytes, hiPSCs, hiPSC-derived GPCs, and hiPSC-derived neurons measured by high-throughput qRT-PCR on the Biomark (Fluidigm). The 35 markers are listed on the x axis, and cell type and donors on the y axis. Results are expressed as the *Z* score of the –ΔC_T_ (C_T_ of *gene of interest* – C_T_ of *GAPDH*). (E) Principal-component analysis including all the 35 markers measured by high-throughput qRT-PCR on the Biomark (Fluidigm). (F) Venn diagram of the genes highly expressed (LogCPM > 5) by resting human primary astrocytes and hiPSC-derived astrocytes based on RNA sequencing data. NSCs, neural stem cells; AGPCs, astrocyte-committed GPCs; SB, SB431542; FGF2, fibroblast growth factor 2; EGF, epithelium growth factor; LIF, leukemia inhibitory factor; CNTF, ciliary neurotrophic factor; HC, healthy control; MS, MS patient; pA, primary astrocytes.

To differentiate astrocytes from hiPSC-derived precursor cells and minimize the concomitant formation of neurons, most authors add FBS to the differentiation medium (Tyzack et al., 2016). However, this serum induces long-term changes in astrocyte gene expression, decreasing the similarity of hiPSC-derived astrocytes to their *in vivo* counterparts (Zhang et al., 2016). Therefore, we aimed at improving generation of astrocytes from hiPSCs without use of serum. First, we induced the neuralization of hiPSCs into neural stem cells (NSCs) using the well-described dual SMAD signaling inhibition (SB431542 with Noggin) (Chambers et al., 2009). NSCs were amplified in the presence of fibroblast growth factor 2 (FGF2) and epidermal growth factor (EGF) for massive cell banking, yielding potentially more than one billion glial precursor cells (GPCs) from two to three million hiPSCs. This method gave rise to a homogeneous cryopreservable population of GPCs, which were used as a starting point for astrocyte differentiation. Concomitant exposure of these cells to leukemia inhibitory factor (LIF) and EGF accelerated the switch toward glial cell differentiation by activation of the JAK-STAT pathway (Bonni et al., 1997). This procedure led to a proliferative population of astrocyte-committed GPCs in only 14 days (Figure 1A).

After 4 weeks of maturation in the presence of ciliary neurotrophic factor (CNTF), these immature cells lost their proliferative capacity and acquired specific astrocyte markers such as GLAST (also known as EAAT1), S100β, and GFAP (Figure 1B). At the end of differentiation, our protocol yielded cell populations composed of more than 94% GLAST+ S100β+ astrocytes similarly to fetal primary astrocyte cultures (Figure 1C). The population generated for each study subject displayed similar morphology and the purity of the culture (percentage of double-positive GLAST+ S100β+ cells) was conserved over the different hiPSC lines. Of note, GFAP was detectable in a variable proportion of astrocytes (Figures 1C and S2).

To characterize the level of maturation of the differentiated astrocytes and their similarity to primary astrocytes, using Fluidigm technology Biomark, we assessed the expression of 35 genes enriched in a given cell type (Figure 1D). Human iPSC-derived astrocytes displayed a pattern similar to human primary astrocytes, which were obtained from three different suppliers, with an enrichment in astrocytic markers compared with hiPSC-derived GPCs. A principal-component analysis revealed that hiPSC-derived astrocytes clustered together with primary astrocytes, clearly discriminating these cell types from hiPSCs, hiPSC-derived GPCs, and hiPSC-derived neurons (Figure 1E). Finally, RNA sequencing analysis of highly expressed genes (LogCPM > 5) revealed that hiPSC-derived astrocytes and primary astrocytes shared 90.3% and 90.6%, respectively, of expressed genes (Figure 1F). These results show that our shortened and simplified protocol produces nearly pure cultures of fully differentiated astrocytes that are very close to primary astrocytes, and this, for all seven study subjects.

To ensure that our mature astrocytes were also functional, we tested for key astrocytic functions. First, astrocytes are known to display waves of calcium transients in response to extracellular stimuli such as glutamate or ATP (Bazargani and Attwell, 2016). We tested for this response in presence of ATP and found that hiPSC-derived astrocytes were indeed able to exhibit calcium transients (Figure S3). Next, we found that culturing differentiating neurons in astrocyte-conditioned medium for a week led to a significant increase in the number of surviving neurons (by 59.5%, p = 0.0167) and to a more complex neurite network (neurite length/neuron increased by 9.6%, p = 0.0424) compared with neural medium (Figures S4A and S4B), therefore demonstrating that our hiPSC-derived astrocytes are fully able to secrete factors supporting neuronal development. Another major function of astrocytes is their capacity to take up glutamate from the environment, thereby preventing synaptic excitotoxicity. We found that astrocytes from all seven study subjects displayed significant glutamate uptake capacity, resulting in a 5-fold increase compared with GPCs (Figure S4C).

### TNF-α and Bovine Serum Exert a High and Relatively Similar Effect on hiPSC-Derived Astrocytic Function and Immune Properties

Astrocytes are well-known responders to inflammatory stimuli (Colombo and Farina, 2016). As tumor necrosis factor alpha (TNF-α) is a classical trigger of reactivity in astrocytes and also plays a major role in neuroinflammation (Becher et al., 2017), we examined the astrocytic response to this cytokine. In addition, FBS may trigger a reactive phenotype in culture (Zhang et al., 2016) so we decided to investigate further the phenotype of hiPSC-derived astrocytes that had been cultured in presence of FBS.

**Figure 2:**
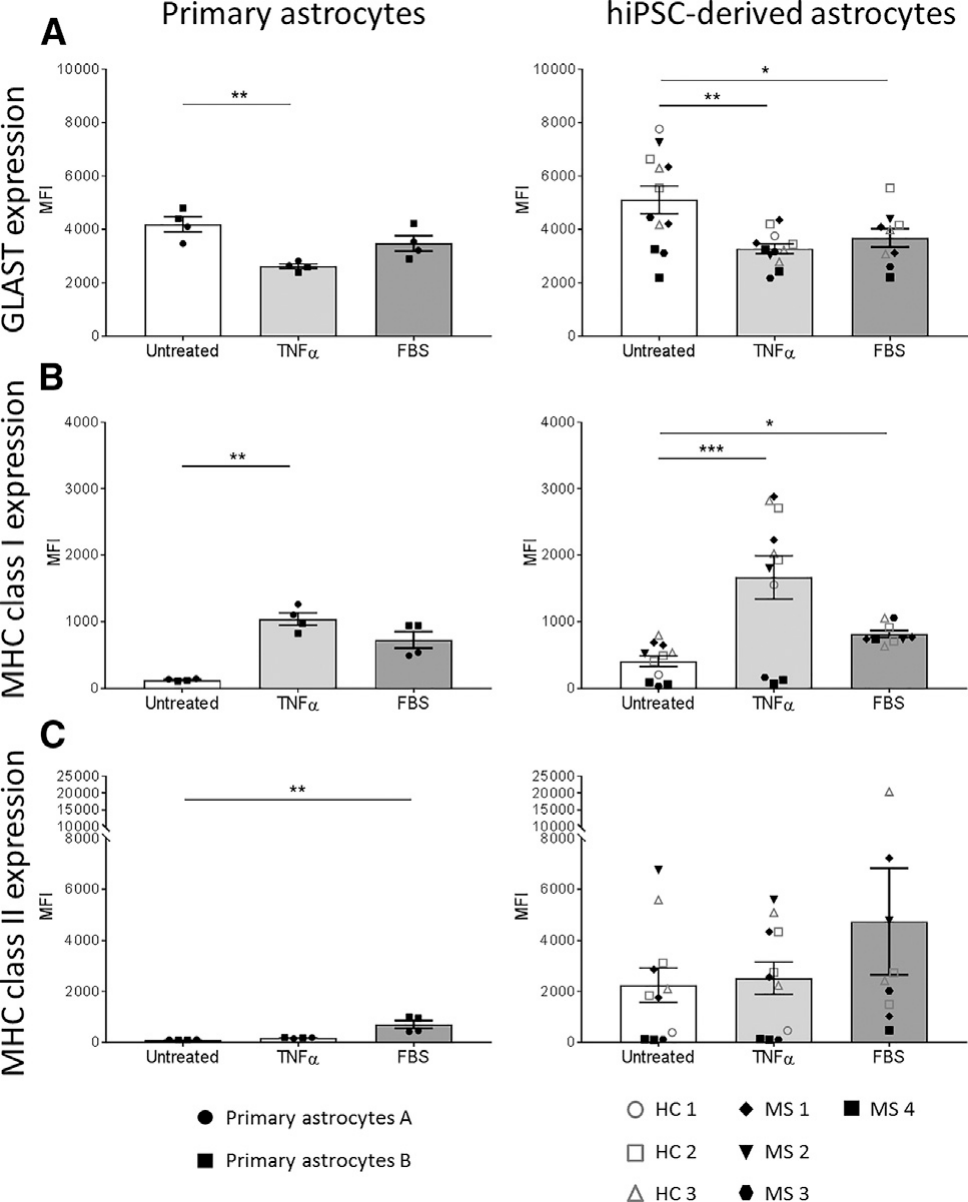
Human iPSC-Derived Astrocytes and Primary Astrocytes Acquire a Reactive Phenotype upon TNF-α and Serum Stimulation Expression of GLAST (A), MHC class I (B), and MHC class II (C) by primary and hiPSC-derived astrocytes was assessed by flow cytometry and in different conditions (untreated, TNF-α [10 ng/mL] and FBS [2%]). Results are expressed as the mean fluorescence intensity (MFI). Each experiment was performed in duplicate and each dot represents the mean results of one or two experiments performed on a given cell line. Bars represent the global mean ± SEM. Only paired data from hiPSC-derived astrocytes were analyzed with a paired Friedman test (ANOVA) followed by a Dunn's multiple comparison test to assess statistical significance as compared with untreated condition (Untreated). Data from primary astrocytes were analyzed with a Kruskal-Wallis test (ANOVA) followed by a Dunn's multiple comparison test to assess statistical significance as compared with untreated condition (Untreated). ^∗^p < 0.05; ^∗∗^p < 0.01; ^∗∗∗^p < 0.001.

We found that, upon TNF-α activation, human astrocytes, both primary and hiPSC-derived, displayed a decreased expression of the glutamate transporter GLAST. Interestingly, FBS also decreased the expression of GLAST by hiPSC-derived astrocytes (p = 0.019) and, to a lesser extent, by primary astrocytes (Figure 2A). We found that, in the presence of either TNF-α or FBS, astrocytes upregulated major histocompatibility complex class I (MHC class I) molecules on their membranes (Figure 2B). By contrast, TNF-α did not increase expression of MHC class II molecules, while FBS did (Figure 2C). These data show that (1) hiPSC-derived astrocytes generated without serum can react to inflammatory stimuli, here TNF-α stimulation; (2) FBS induces the same phenotype as TNF-α even though with a lesser intensity; and (3) hiPSC-derived astrocytes behave the same way as primary cells.

### Serum Induces Major Transcriptomic Changes in the Metabolism and Response to Stimuli

**Figure 3:**
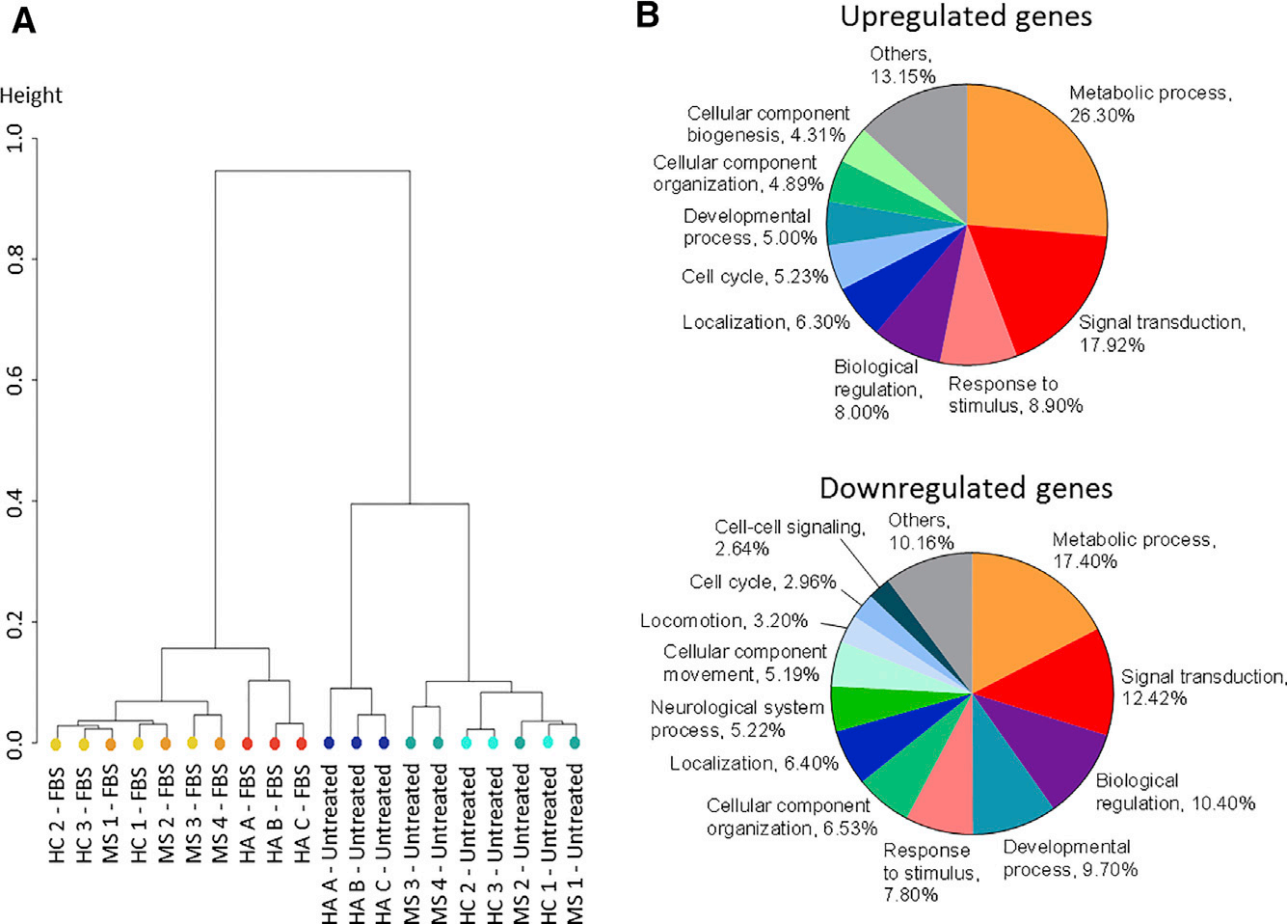
Serum Induces Profound Transcriptomic Changes in Human Astrocytes RNA sequencing analysis of primary and hiPSC-derived astrocytes untreated and stimulated with FBS. The analysis was performed on one hiPSC line per donor and three different primary astrocytes, each from a different supplier. (A) Unsupervised clustering dendrogram of astrocyte samples based on significantly up- and downregulated genes (false discovery rate [FDR] < 0.05) in FBS conditions compared with untreated conditions. (B) Classification by biological processes (gene ontology, PANTHER classification system) of upregulated genes and downregulated genes in FBS conditions compared with untreated conditions (FDR < 0.05).

Since FBS appears to affect the phenotype of astrocytes in the same way as TNF-α, its addition to culture medium may therefore bias any study focusing on astrocyte responses to inflammation. Yet, the majority of hiPSC-derived astrocyte culture protocols include FBS. Thus, we decided to investigate on a broader scale its impact on astrocytes. Therefore, we performed an RNA sequencing analysis of primary and hiPSC-derived astrocytes, either untreated or exposed to FBS. This analysis revealed that all astrocytes treated with serum clustered together regardless of their origin and with a long orthogonal distance as compared with the untreated groups (Figure 3A). In other terms, the presence or not of FBS was much more discriminating than the source of astrocytes, either primary or hiPSC-derived. Numerous pathways were affected (either up- or downregulated) by the presence of FBS. Changes in expression of mRNA coding for metabolic processes, signal transduction, biological regulation, and responses to stimuli accounted for 61% and 47% of the up- and downregulated genes, respectively (Figure 3B).

Taken together, these data suggest that addition of serum to astrocytic cultures profoundly affect their transcriptome, and point to the validity of our serum-free protocol as an unbiased model to examine the role of astrocytes in response to inflammation. Thus, in subsequent experiments, we used only hiPSC-derived astrocytes or human primary astrocytes that had been cultured without serum.

### Multiple Sclerosis-Associated Cytokines Trigger Specific Transcriptomes in Human Astrocytes

**Figure 4:**
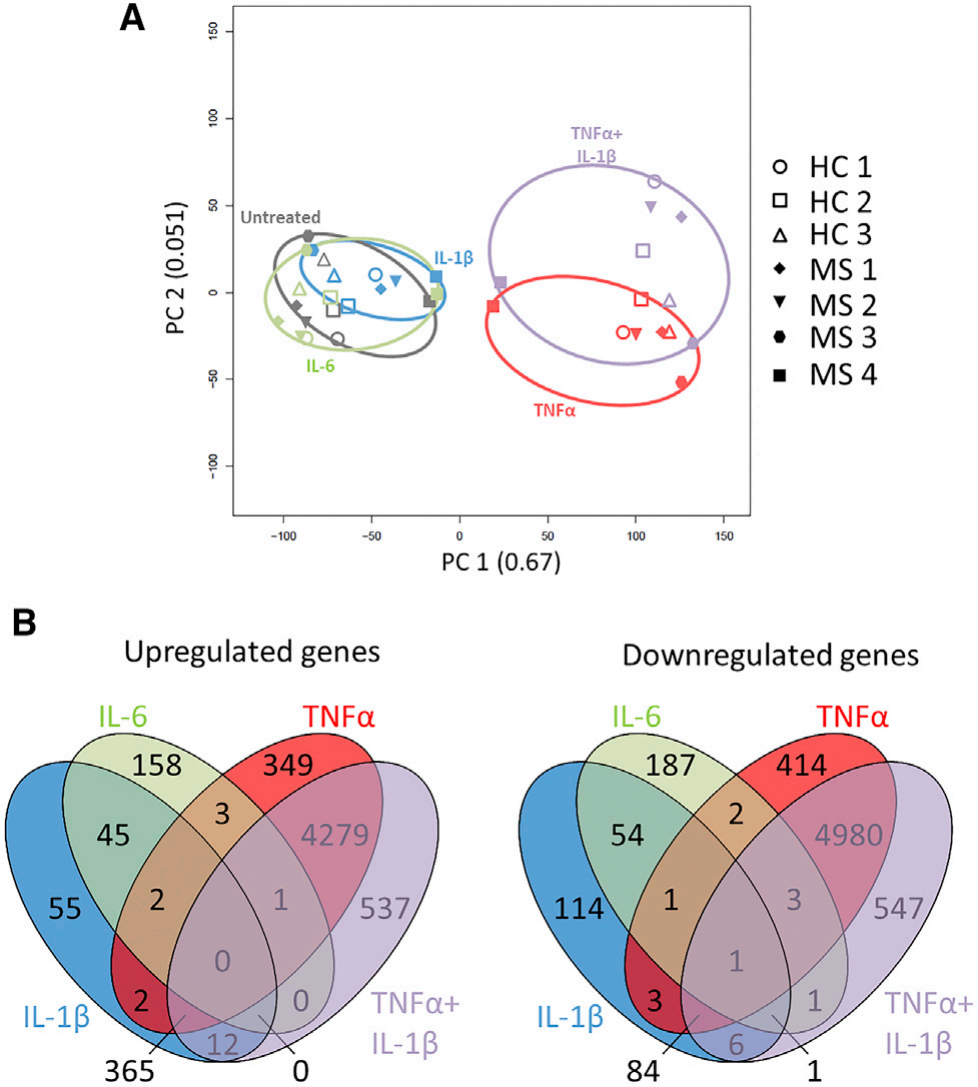
MS-Associated Cytokines Induce Distinct Transcriptomic Changes RNA sequencing analysis of hiPSC-derived astrocytes untreated and stimulated with IL-6, IL-1β, TNF-α, or IL-1β + TNF-α. The analysis was performed on one hiPSC line per donor. (A) Principal-component analysis plotting hiPSC-derived astrocytes identified by donor and condition of stimulations and based on up- and downregulated genes (FDR < 0.05) from stimulated conditions compared with untreated conditions. (B) Venn diagrams of significantly upregulated and downregulated genes (FDR < 0.05) from stimulated conditions compared with untreated conditions.

MS is a prototypical neuroinflammatory disease in which astrocytes are in contact with microglia and pro-inflammatory infiltrating immune cells invading the brain (Mayo et al., 2012). Previous studies linked lipopolysaccharide (LPS)-triggered neuroinflammation to a harmful astrocyte phenotype associated with a clear transcriptomic profile (Zamanian et al., 2012), but others have highlighted the dual role of astrocytes in the experimental autoimmune encephalomyelitis (EAE) model of MS, depending on the stage of the disease (Mayo et al., 2014). As these data hint at a profound impact of the neuroinflammatory environment on astrocyte phenotype, we sought to investigate the impact of three crucial cytokines in MS, i.e., interleukin-1β (IL-1β), TNF-α, and IL-6, at a transcriptomic level (Becher et al., 2017). First, a principal-component analysis revealed the strong effect of TNF-α on astrocyte transcriptome as both conditions containing this cytokine (TNF-α alone and TNF-α + IL-1β) clustered closer than the three other conditions (Figure 4A). Of note, we did not identify different clustering profiles between healthy controls and MS patients. Among the three tested cytokines, TNF-α was by far the one having the most potent effect on astrocytes as it upregulated 5,001 and downregulated 5,488 genes, with only 7% of the upregulation and 2% of the downregulation being shared with the two other cytokines (false discovery rate [FDR] < 0.05; Figure 4B). Although IL-6 had a lesser effect on astrocytes, upregulating “only” 209 and downregulating 250 genes, it nevertheless had a specific action with only 24% of the upregulated and 25% of the downregulated genes being shared with the two other cytokines (Figure 4B). Among the three cytokines, IL-1β was the one with the least specific action since, of the 481 upregulated genes, as many as 86% were shared with one or the other cytokine, whereas of the 264 downregulated genes, 55% were shared with TNF-α or IL-6 (Figure 4B). Interestingly, the co-stimulation of astrocytes by IL-1β and TNF-α led to the modulation of a total of 1,104 additional genes (Figure 4B), highlighting that even small changes in the precise composition of the CNS milieu during neuroinflammation may affect astrocyte phenotype and functionality.

### Inflammatory Cytokines Specifically Modulate Astrocytic Secretion of Cytokines and Remyelination Factors

Next, as astrocytes are pivotal in the immune response of the CNS and can relay inflammation, we looked at the secretion of a large panel of cytokines associated with MS in the supernatant of reactive astrocytes. Granulocyte-macrophage colony-stimulating factor (GM-CSF), TNF-α, IL-1β, IL-1α, and IL-6 were measured for their implication in MS immunopathology (Becher et al., 2017), and IL-12 and IL-23 for their role in T helper type 1 (Th1) and Th17 differentiation, respectively, as these two T cell subsets are related to MS (Hohlfeld et al., 2015). Finally, type I interferons (IFN-α and -β) and IL-10 were investigated for their protective role in MS (Kwilasz et al., 2015, Prinz and Knobeloch, 2012).

**Figure 5:**
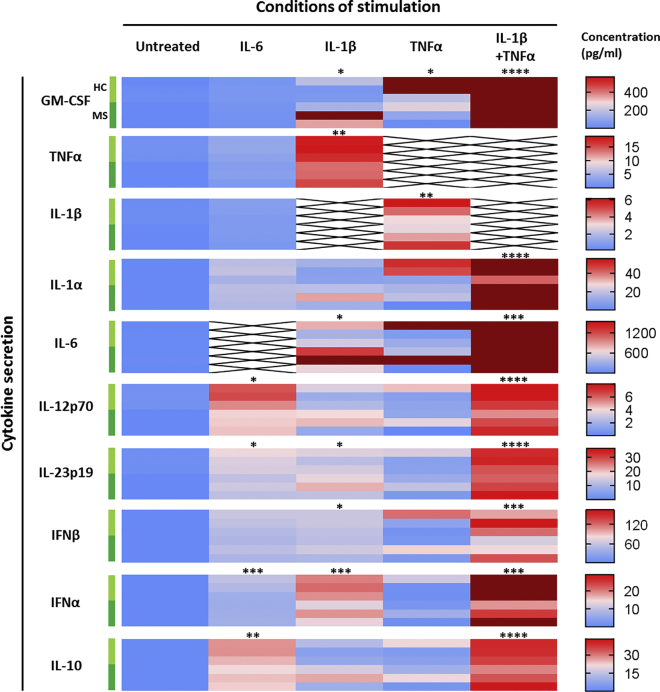
Distinct Cytokine Stimulation of hiPSC-Derived Astrocytes Triggers Specific Secretion Profiles Cytokine secretion was measured in the supernatant of hiPSC-derived astrocyte cultures following stimulation with MS-associated cytokines for 5 days and compared with untreated cells (Untreated). Concentrations were measured by multiplexed bead-based immunoassay (Luminex). Data are presented as a heatmap of concentrations (pg/mL) going from 0 to the highest value measured for each cytokine after outlier exclusion (calculated with the ROUT method on GraphPad Prism 7, in dark red on the heatmaps). Conditions of stimulation are stacked in columns and cytokines analyzed in lines. For each cytokine analyzed, each of the six horizontal lines represents one study subject, starting with the three HCs (light green) then three MS patients (dark green). Each result represents the mean of two replicates of one experiment performed on one hiPSC line. Data were analyzed with a paired Friedman test (ANOVA) followed by a Dunn's multiple comparison test to assess statistical significance compared with the untreated condition (^∗^p < 0.05; ^∗∗^p < 0.01; ^∗∗∗^p < 0.001; ^∗∗∗∗^p < 0.0001).

We found that at basal levels, resting astrocytes only secreted IL-6 in low amounts (mean = 5.3 pg/mL); all other cytokines were below the limit of quantification. Interestingly, each of the cytokines used as stimuli triggered the production of various other cytokines leading to a unique secretion profile for each activation stimulus (Figure 5). Both TNF-α and IL-1β triggered a strong inflammatory phenotype in astrocytes although IL-1β was a more potent activator than TNF-α, leading to pan-activation of astrocytes, characterized by large amounts of IL-6 (mean = 1,301.8 pg/mL) and GM-CSF (mean = 214.4 pg/mL), associated with significant production of TNF-α (mean = 16.1 pg/mL) and IL-23 (mean = 15.4 pg/mL) as well as secretion of some cytokines protective in MS, type I interferons (IFN-β mean = 56.8 pg/mL and IFN-α mean = 17.4 pg/mL). TNF-α alone triggered a larger secretion of GM-CSF (mean = 335.6 pg/mL), which was accompanied by significant secretion of IL-1β (mean = 4.3 pg/mL) only, displaying a more limited activation than observed with IL-1β. Interestingly, co-stimulation with TNF-α and IL-1β resulted in a huge synergistic effect, enhancing production of both pro- and anti-inflammatory mediators. Compared with IL-1β only, co-stimulation with IL-1β and TNF-α induced a massive increase in the secretion of GM-CSF (13.1-fold increase), IL-6 (10.1-fold increase), IL-10 (2.7-fold increase), IL-12 (2.4-fold increase), IL-23 (2.0-fold increase), IFN-β (1.9-fold increase), and IFN-α (1.7-fold increase).

IL-6 also triggered broad secretion of cytokines but in smaller amounts than seen with IL-1β and, interestingly, the most induced cytokines were IFN-β (mean = 51.8 pg/mL) and IL-10 (mean = 24.5 pg/mL), followed by IL-1α (mean = 17.3 pg/mL), IL-23 (mean = 14.4 pg/mL), IFN-α (mean = 7.5 pg/mL), and finally IL-12 (mean = 5.1 pg/mL) (Figure 5). This particular profile suggests that IL-6-activated astrocytes may have a regulatory role.

Taken together, these data underline the wide range of secretory phenotypes that astrocytes acquire depending on their environment and, as a result, the distinct roles astrocytes can play in neuroinflammation in general and in MS in particular.

**Figure 6:**
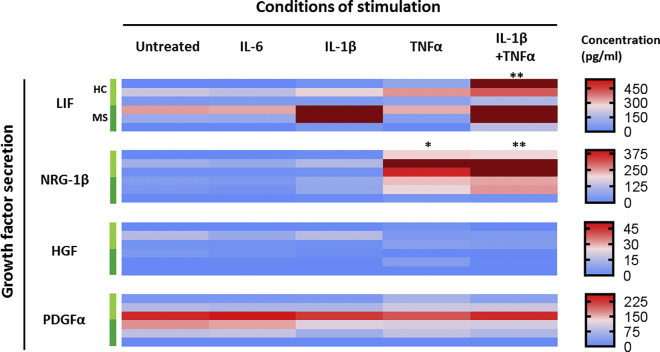
Inflammatory Cytokines Specifically Modulate Astrocytic Secretion of Growth Factors Associated with Remyelination Secretion of remyelinating-associated growth factors by hiPSC-derived astrocytes was analyzed following stimulation with inflammatory cytokines and compared with untreated cells (Untreated). Concentrations were measured by multiplexed bead-based immunoassay (Luminex). Conditions of stimulation are stacked in columns and growth factors analyzed in lines. For each growth factor analyzed, each of the six lines represents one study subject, starting with the three HCs (light green) then three MS patients (dark green). Each result represents the mean of two replicates of one experiment performed on one hiPSC line. Data were analyzed with a paired Friedman test (ANOVA) followed by a Dunn's multiple comparison test to assess statistical significance as compared with untreated condition (^∗^p < 0.05; ^∗∗^p < 0.01). NRG-1β, neuregulin-1β.

In MS, the inflammatory phases are followed by a partial resolution of the lesions and repair of the CNS tissue including remyelination of axons. This process is mediated in part by astrocytes through secretion of growth factors (Moore et al., 2011). We examined whether inflammatory mediators were able to modify the secretion of some factors involved in the proliferation of oligodendrocyte precursor cells, i.e., PDGFα and HGF, or the differentiation of oligodendrocyte precursor cells into myelinating oligodendrocytes, i.e., LIF and neuregulin-1β (Domingues et al., 2016, Yan and Rivkees, 2002). Interestingly, contrasting with cytokines, we found that the four growth factors were constitutively secreted in resting conditions with, respectively, 98.8, 3.9, 117.6, and 32.1 pg/mL (Figure 6). After stimulation, TNF-α led to an 8.8-fold increase in neuregulin-1β secretion (p = 0.0186). IL-1β increased LIF secretion by 2.2-fold, but this was not significant (p = 0.5765). Co-stimulation with IL-1β and TNF-α raised the level of LIF secretion by 2-fold compared with IL-1β alone, but induced only a slight upregulation of neuregulin-1β as compared with TNF-α alone. Stimulation with IL-6 did not modulate the secretion of pro-myelinating growth factors. Interestingly, these data suggest that a strong pro-inflammatory environment somewhat favors repair of the CNS, at least as far as astrocytes are concerned.

## Discussion

As the interest in the role of astrocytes in neuroinflammation is increasing, several protocols to generate astrocytes from hiPSCs have been published. However, to the best of our knowledge, astrocytes differentiated from hiPSCs from MS patients have not been reported. Here we established a serum-free protocol that consistently yields a nearly pure population of astrocytes from GPCs in only 6 weeks. Instead of using serum to promote GPC differentiation into astrocytes, we added LIF to GPC medium to trigger the glial switch, shifting GPCs' potential from neurogenic to gliogenic. We validated our protocol on 13 hiPSC lines, 5 from healthy donors, and 8 from MS patients, thereby demonstrating the robustness and reliability of our protocol to generate human functional astrocytes. As the latter cells cannot be identified by a single marker, most authors describing hiPSC-derived astrocytes use three or four different markers to assess their identity (Chandrasekaran et al., 2016). Here, to optimally characterize the cells generated, we used a combination of 35 markers of different cell lineages, which revealed an enhanced expression of astrocytic genes, a downregulation of precursor genes, and low levels of neuronal and hiPSC markers (Figure 1). Most importantly, our hiPSC-derived astrocytes exhibited a phenotype similar to primary human astrocytes.

Among the protocols differentiating astrocytes from hiPSCs, only a handful of them demonstrated the reactive potential of these cells (Lundin et al., 2018, Roybon et al., 2013, Santos et al., 2017, Tcw et al., 2017). They show that these cells are able to react to inflammatory stimuli; however, most use serum in the culture medium to differentiate GPCs into astrocytes. Yet, serum induces some features of reactive astrocytes as opposed to astrocytes cultured in serum-free conditions (Zamanian et al., 2012, Zhang et al., 2016), which is the reason why we developed this serum-free approach to obtain astrocytes from hiPSCs. To ensure that our differentiation protocol generates resting astrocytes, we compared their phenotype to the one of FBS-treated cells. We show that FBS-treated astrocytes, either differentiated from hiPSCs or primary ones, acquire a reactive phenotype similar to the one displayed after TNF-α stimulation (Figure 2). Interestingly, FBS triggers strong changes in the transcriptome, in particular in genes associated with basal functions (metabolism processes and biological regulation), response to stimuli, and signal transduction. These data demonstrate that FBS is a potent modifier of astrocyte responses to their environment, and suggest that if one wants to study the response of astrocytes to inflammatory stimuli, using astrocytes that have been cultured in the presence of FBS may bias the results. Hence, we believe that our serum-free model will be a useful tool in the study of MS and other neuroinflammatory diseases.

As MS is a prototypic neuroinflammatory disease, we used our model to investigate the role of activated astrocytes in this pathology. In MS, immune cells infiltrate the CNS parenchyma from the periphery, which leads to destruction of the myelin sheath and damages to axons. There is growing evidence that CNS cells participate actively to this process, astrocytes emerging as potential major contributors to MS (Correale and Farez, 2015). So far, the putative role of astrocytes has been studied mostly in mice (EAE model), not in human MS patients and translation of this knowledge from rodent could not necessarily be applied to human (Baker and Amor, 2015, Tarassishin et al., 2014).

Here, the stimulation of hiPSC-derived astrocytes with MS-related cytokines leads to several interesting findings. First, TNF-α induces major transcriptomic changes in astrocytes with many more modulated genes than IL-1β and IL-6, confirming the importance of this cytokine as a potent stimulus of astrocytes. Nevertheless, all three cytokines exhibit a specific signature, regulating unique genes that remain unaffected by the two other cytokines. In addition to the unique profiles triggered by the three cytokines studied here, co-stimulation by IL-1β and TNF-α induces important changes in the transcriptome, demonstrating a synergistic rather than a simple additive effect of the two cytokines. We then assess whether the diversity observed at the transcriptomic level was present also at the proteic level. We find that both IL-1β and IL-6 induce a pan-activation of astrocytes resulting in the secretion of a large number of the cytokines studied. TNF-α increases the secretion of GM-CSF, IL-1β, and IL-6. TNF-α also induces the expression of MHC class I molecules on the surface of astrocytes, making them potential targets for CD8+ T cells. Notably, others and ourselves have shown that CD8+ T cells are suspected pathogenic effectors in MS (Babbe et al., 2000, Jilek et al., 2007, Jilek et al., 2008). In addition, data from mouse models show that astrocyte-specific CD8+ T cells can mediate spontaneous relapsing-remitting CNS autoimmunity (Huseby et al., 2015). However, TNF-α also significantly increases the secretion of the growth factor neuregulin-1β, as well as LIF when added in combination with IL-1β. These data suggest that, depending on the surrounding conditions, TNF-α may have a dual effect on astrocytes; pro-inflammatory and pro-remyelination. The complex role of TNF-α in MS is well illustrated by the fact that anti-TNF-α antibodies have opposite roles in mice and humans, improving EAE, but worsening human MS (Becher et al., 2017).

Among the cytokines produced by astrocytes, the most striking ones are GM-CSF and IL-6. Both cytokines are deregulated in the blood of MS patients (Hartmann et al., 2014, Ireland et al., 2015) and have been demonstrated to be key mediators of neuroinflammation (Becher et al., 2017, Giralt et al., 2013). So far, GM-CSF was shown to be produced mainly by T cells (Becher et al., 2017) and B cells, to a lesser extent (Mathias et al., 2016). Interestingly, our study points out an important capacity of astrocytes to produce high quantities of this MS-essential cytokine, raising interesting questions for MS immunopathogenesis. Other authors have already shown that IL-6 was produced by astrocytes in MS lesions (Schonrock et al., 2000). Our study confirms this finding and also puts in evidence the wide reactivity induced by IL-6, highlighting the specific link between this cytokine and astrocytes.

In mice, recent in-depth characterization of reactive astrocytes has demonstrated that neuroinflammation triggered by LPS injection in the brain led to a neurotoxic phenotype named A1 as opposed to an A2 phenotype, protective in ischemia (Liddelow and Barres, 2017, Liddelow et al., 2017). Our findings suggest that this classification may not account for the complexity of human astrocytic response in the context of neuroinflammation.

Of note, we observe no significant differences between the profiles of astrocytes derived from healthy controls and from MS patients. These results are in agreement with the observation that genetic risks associated with MS are mostly linked to the regulation of the immune response rather than to CNS cell-related genes (Sawcer et al., 2011). Based on this view, this dysregulated immune response is considered to be the part of the pathogenesis specific to MS, whereas the activation of glial cells, astrocytes, and microglia, reflects more a normal response of these cells to inflammatory stimuli and is not a mechanism specific to MS. However, our study demonstrates that different inflammatory stimuli lead to particular profiles of human astrocytes as seen both at the transcriptome and at the cytokine secretion level. These data demonstrate that the development of hiPSC-derived astrocytes may be a crucial tool to better understand the complex role played by these cells in neuroinflammatory diseases.

In conclusion, we developed a serum-free method generating human astrocytes to study their role in neuroinflammation. We used this tool to investigate the profiles of astrocytes after stimulation with MS-associated cytokines, and demonstrated that this model may be relevant as per the detailed study of the role of astrocytes in MS. Given the differences existing between rodent and human astrocytes and the failures in translation of potential drugs from mice to humans (Baker and Amor, 2015), we anticipate the value of such a model as a screening tool for development of future therapies in neuroinflammatory diseases.

## Experimental Procedures

### Reprogramming, Culture, and Characterization of Human iPSCs

Three healthy controls and two relapsing-remitting MS (RR-MS) patients were enrolled in Lausanne, Switzerland ([donor: age/sex]: HC1: 27/F; HC2: 50/M; HC3: 49/F; MS3: 21/F; MS4: 31/F). Two other RR-MS patients were enrolled in Nantes, France (MS1: 15/M; MS2: 17/F). All subjects gave their written informed consent according to institutional review board guidelines (consent form 107/13 for Lausanne subjects and OFSEP consent form for Nantes subjects). The blood of all four MS patients was drawn at the time of their first relapse, when all four were not on disease-modifying treatment (DMT) yet. Subsequently all four MS patients were confirmed to suffer from RR-MS and three of them were put on DMT consisting of natalizumab, fingolimod, or dimethylfumarate. Peripheral blood mononuclear cells (PBMCs) from study subjects were isolated as described previously (Jilek et al., 2008) and frozen until use. For reprogramming into hiPSCs, erythroblasts were amplified and sorted from PBMCs before nucleofection with episomal plasmids encoding for OCT4, shRNA-p53, SOX2, KLF4, L-Myc, and Lin28 (Okita et al., 2011). Cells were plated on Matrigel-coated plates (Corning) in erythroblast medium and cultured in ReproTeSR medium (STEMCELL) until hiPSC clones started to appear (see details in Supplemental Information). Donor-specific hiPSC lines were expanded in StemMACS iPSC-Brew XF medium (Miltenyi) and characterized for pluripotency and differentiation capacity (see details in Supplemental Information). Karyotype analysis was performed by the Constitutional genetics laboratory of the University Hospital of Lausanne.

### Differentiation of hiPSC-Derived Neurons and Astrocytes

All media compositions are detailed in the Supplemental Information (Table S1).

Human iPSCs were first differentiated into NSCs as described previously (Boissart et al., 2013). In brief, iPSC medium was changed to Neural Induction medium in the presence of Y-27632 (10 μM; Miltenyi). Cells were mechanically detached using a cell scraper and cultured in suspension for 6 hr before plating on poly-L-ornithine/laminin (PO/L)-coated plates. Medium was changed the next day and every other day for 10 days until neural rosettes appeared in the culture.

For neuronal differentiation, medium was changed to Neural Expansion medium. After 3 days, rosettes were manually selected and replated on new PO/L-coated plates in the presence of Y-27632 (10 μM). When cells reached confluence, neural rosettes were dissociated using trypsin (BioConcept) and replated at 100,000 cells/cm^2^ in PO/L-coated flasks. Neural precursor cells (NPCs) were expanded for 6 passages before cell banking in liquid nitrogen. For final neuronal differentiation, NPCs were thawed and plated at 50,000 cells/cm^2^ on PO/L-coated plates in neural medium. Medium was changed every 2 days and supplemented with CultureOne supplement (Gibco) 2× for the first 7 days only. CultureOne supplement was removed after the first week and medium was changed every 3–4 days for 3 weeks.

For astrocyte differentiation, medium of D10 neural rosettes was changed to Glial Expansion medium. Rosettes were picked up as described for NSCs except that we used Glial Expansion medium to obtain GPCs, amplified for at least eight passages before massive cell banking in liquid nitrogen. For final astrocyte differentiation, GPCs were thawed and plated on Matrigel-coated flasks at 50,000 cell/cm^2^ in Astrocyte Induction medium for 14 days and passaged when cultures reached confluence. At this point, the cell population had to be completely homogeneous. Finally, cells were cultured for 4 weeks in Astrocyte medium supplemented with CNTF (20 ng/mL, PeproTech) to obtain mature astrocytes. During this transition period, cells were passaged when confluent or when GPCs started to proliferate too much (formation of dense cell clusters). Astrocytes started to emerge in the culture from 5 to 7 days and slowly proliferated while the remaining GPCs continued to differentiate into astrocytes. Appearance of a small proportion of neurons is normal during this step, but these cells die during the passages allowing to obtain a culture highly enriched in astrocytes. After 4 weeks, mature astrocytes were cultured in Astrocyte medium without CNTF. Each hiPSC line was differentiated once into GPCs, and the differentiation of GPCs into astrocytes was conducted two to three times for each hiPSC line to ensure robustness of the protocol.

### Human Primary Astrocyte Cell Culture

Human primary astrocytes were bought from three different suppliers (A, Thermo Fisher Scientific; B, ScienCell; C, Cell Applications) and amplified according to the manufacturer's instructions. For experiments, cells were cultured for at least 2 weeks in Astrocyte medium without or with FBS depending on the experiment.

### Immunocytochemistry and Flow Cytometry

Cells were stained for immunocytochemistry and flow cytometry as described previously (Perriard et al., 2015). In brief, for immunocytochemistry, cells were fixed with 4% paraformaldehyde, blocked for 1 hr, then incubated overnight with primary antibodies. The next day, cells were incubated with secondary antibodies before counterstaining with DAPI and slides were mounted with ProLong Diamond Antifade medium (Life Technologies). For flow cytometry staining, cells were first stained with violet Live/Dead (Life Technologies) before staining for extracellular markers with labeled antibodies. For intracellular staining, cells were fixed and permeabilized before adding primary antibodies, then washed and stained with labeled secondary antibodies. See Supplemental Information for detailed methods (Table S2).

### Gene Expression

Cells were lysed in RLT Plus buffer (QIAGEN) and RNA was extracted using the RNeasy Plus Mini Kit (QIAGEN). After reverse transcription with the Reverse Transcription Kit (Fluidigm), cDNA preamplification was carried out as recommended by the supplier (Fluidigm). Finally, samples and primers (Delta Gene assays from Fluidigm) were loaded on a chip according to manufacturer's instructions and the chip was prepared with the IFC controller MX. Data were acquired with the Biomark system (Fluidigm). Results were normalized to endogenous control GAPDH and expressed using the ΔC_T_ method.

### RNA Sequencing

RNA sequencing data were generated on the Illumina HiSeq platform and read aligned to human hg19 genome. Statistical analysis was performed for genes in R (R version 3.4.3). Genes with low counts were filtered out according to the rule of one count per million (CPM) in at least one sample. Library sizes were scaled using trimmed mean of M values normalization and log-transformed into CPM (EdgeR package version 3.20.8) (Robinson et al., 2010). Differential expression was computed with the limma-trend approach (Ritchie et al., 2015) by fitting the compared samples into one linear model. Moderated F test was applied and *post-hoc* classification performed. The adjusted p value was computed by the Benjamini-Hochberg method, controlling for FDR. The library construction, sequencing, and statistical analysis were performed by the Genomic Technologies Facility of the University of Lausanne.

### Astrocyte Stimulation

On day 0 of an experiment, cells were washed once with Astrocyte medium then incubated at 37°C with stimulus-supplemented medium (cytokines or FBS) for the time indicated in the figure legends. Stimulations included TNF-α (10 ng/mL, R&D Systems), IL-6 (100 ng/mL, Miltenyi), IL-1β (10 ng/mL, Miltenyi), and FBS (2%).

### Bead-Based Immunoassays

After stimulation of astrocytes for 5 days, supernatants were harvested, centrifuged for 10 min at 2,000 × *g* and stored at −20°C until use. All cytokine and growth factor concentrations were measured using multiplex bead-based immunoassays (Affymetrix and R&D Systems, detailed in Supplemental Information) according to the manufacturer's instructions. Data were acquired with Bio-Plex 200 reader (Bio-Rad) and analyzed using the Bio-Plex Manager software (version 5.0).

### Statistical Analysis and Heatmap Representations

Statistical analyses were performed with GraphPad Prism software version 7.03. Paired non-parametric Wilcoxon test was performed to compare groups two by two. The differences among three groups or more were tested using the paired non-parametric Friedman test, followed by *post-hoc* Dunn's multiple comparison test if significant. A p value <0.05 was considered significant. Principal-component analyses were performed using the prcomp module of RStudio software version 0.99.902. For heatmap representations (Figures 5 and 6), zero was set as minimum and the highest value was set as maximum after exclusion of outliers (represented in dark red on the graphs) determined by ROUT method.

## Author Contributions

S.P. designed the study, performed the experiments, analyzed the data, and wrote the manuscript. A.M. designed the study, discussed the results, and revised the manuscript. G.P., M.C., N.J., N.M., and C.M. performed the experiments. L.E.K. and A.L.P. provided the technique for reprogramming erythroblasts into hiPSCs and revised the manuscript. M.S. and D.-A.L. enrolled study subjects and revised the paper. N.D. contributed to the design of the study and revised the manuscript. R.D.P. designed the study, discussed the results, revised the manuscript, and supervised the whole study.

## Accession Numbers

RNA sequencing data have been deposited in the NCBI's GEO database under accession number GEO: GSE120411.

## Supplemental Information

Document S1. Supplemental Experimental Procedures, Figures S1–S4, and Tables S1 and S2

Document S2. Article plus Supplemental Information
