# Supplementary material for: Human Induced Pluripotent Stem Cell-Derived Astrocytes Are Differentially Activated by Multiple Sclerosis-Associated Cytokines
Source: Stem Cell Reports. 2018 Oct 25;11(5):1199–210. doi: 10.1016/j.stemcr.2018.09.015 (PMC6234919; doi:10.1016/j.stemcr.2018.09.015)
Supplement: Document S1. Supplemental Experimental Procedures, Figures S1–S4, and Tables S1 and S2 [file mmc1.pdf]

Stem Cell Reports, Volume *11*

## Supplemental Information

# Human Induced Pluripotent Stem Cell-Derived Astrocytes Are Differentially Activated by Multiple Sclerosis-Associated Cytokines

**Sylvain Perriot, Amandine Mathias, Guillaume Perriard, Mathieu Canales, Nils Jonkmans, Nicolas Merienne, Cécile Meunier, Lina El Kassar, Anselme L. Perrier, David-Axel Laplaud, Myriam Schluep, Nicole Déglon, and Renaud Du Pasquier**

## SUPPLEMENTAL INFORMATION

### SUPPLEMENTAL FIGURES

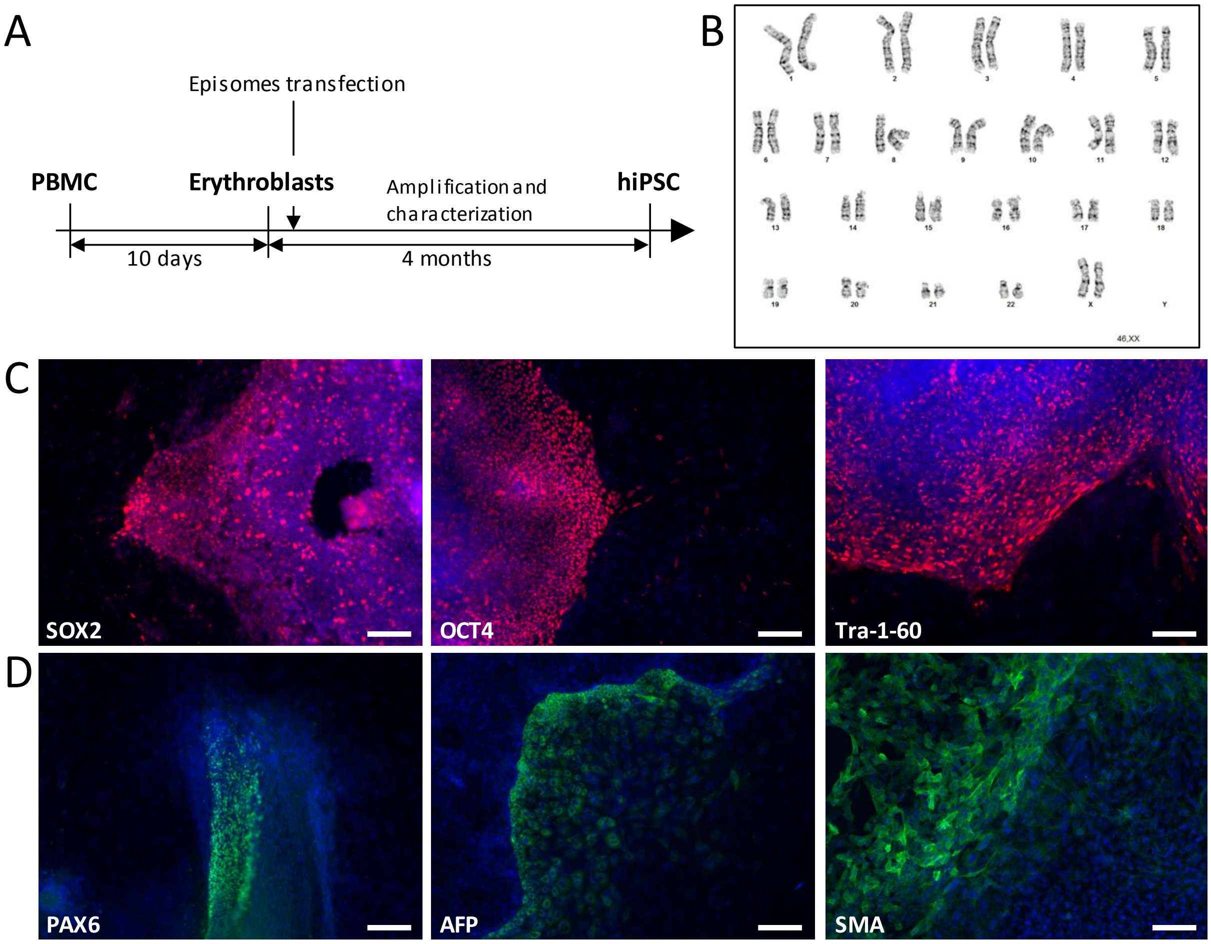


**Figure S1: hiPSC reprogramming and characterization**

(A) Schematic representation of hiPSC reprogramming from PBMC.
(B) Representative picture of a normal hiPSC karyotype showing that no abnormality was acquired during the reprogramming phase.
(C) Full pluripotency was monitored by immunofluoresence staining of pluripotency markers (SOX2, OCT4, Tra-1-60) (in red). Cells were counterstained with DAPI (in blue). Scale bars: 200µm. Pictures were acquired with inverted microscope Axiovision Observer.Z1.
(D) After differentiation of hiPSC into embryoid bodies (EB), the capacity of hiPSC to form the three embryonic germ layers was assessed by IF staining with one marker per germ layer (green): ectoderm (PAX6), endoderm (AFP) and mesoderm (SMA). Cells were counterstained with DAPI (blue). Scale bars: 200µm. Pictures were acquired with inverted microscope Axiovision Observer.Z1.

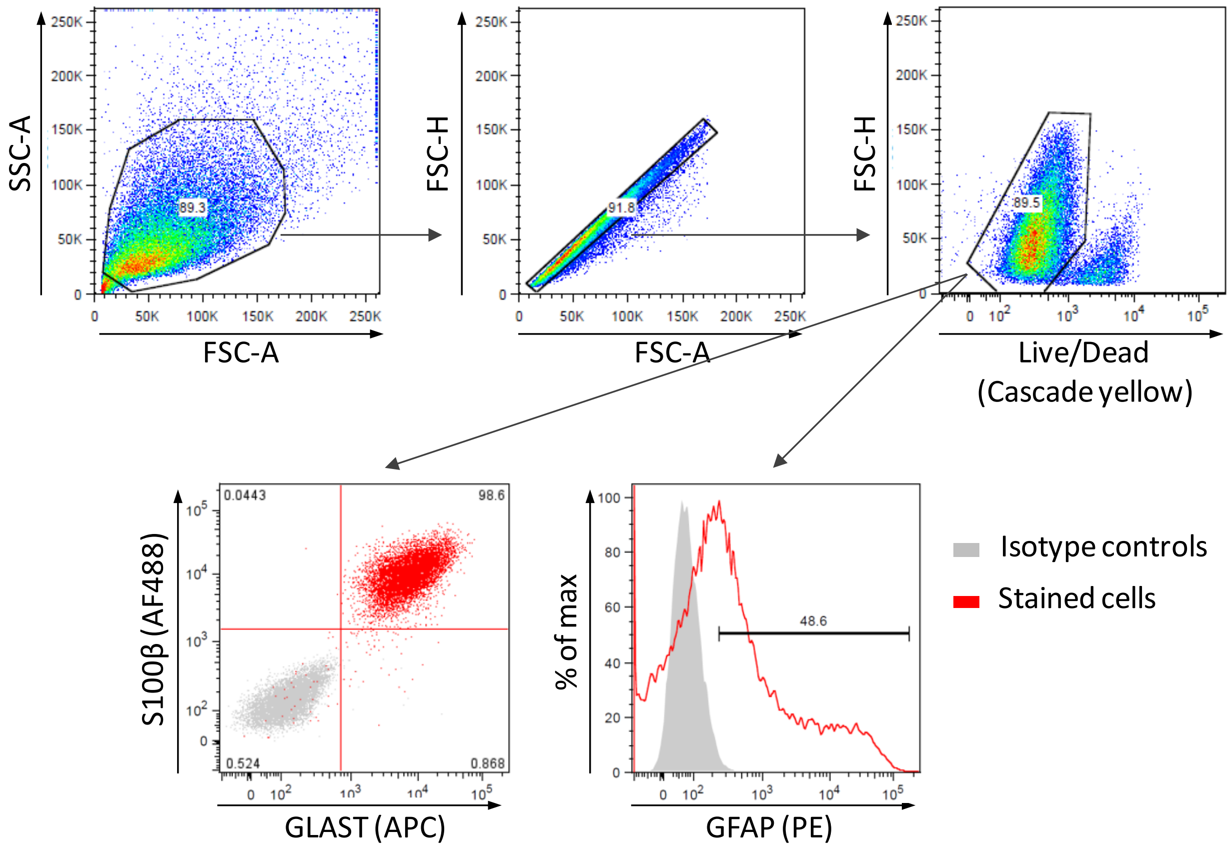


**Figure S2: Gating strategy in the flow cytometry experiments**

The gating strategy is shown for HC 3 donor-derived astrocytes. These data are representative **of** all seven study subjects. Cells were stained such as detailed in the Experimental procedures section. Dead cells were exclude**d** using Live/dead marker. Data were acquired on a LSRII flow cytometer and analyzed using FlowJo Software.

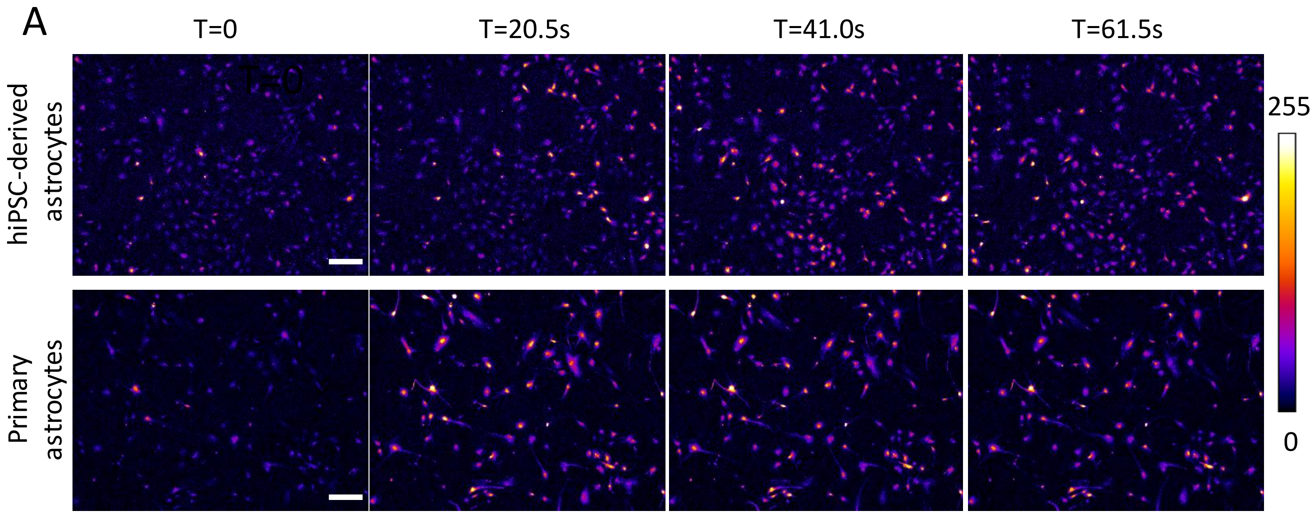


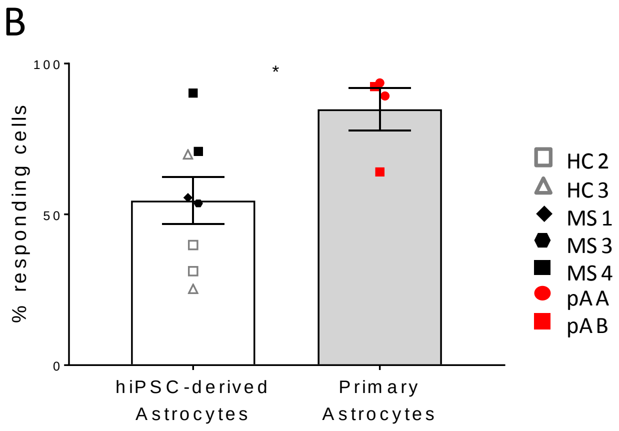


**Figure S3: Human iPSC-derived astrocytes display waves of calcium transients**

(A) Representative pictures of time-lapse calcium transients responses from astrocytes derived from healthy donor HC3 and primary astrocytes B. Pictures are pseudocolorized depending on fluorescence intensity (from purple to yellow). Scale bars: 100µm. Pictures were acquired with inverted microscope Axiovision Observer.Z1.

(B) Percentage of cells displaying calcium transients calculated using ImageJ for data processing.

Each dot represents the mean results of one experiment performed in duplicate for each donor. Bars represent the global mean ± SEM. Mann-Withney test was performed to assess statistical significance ($p<0.05$: *).

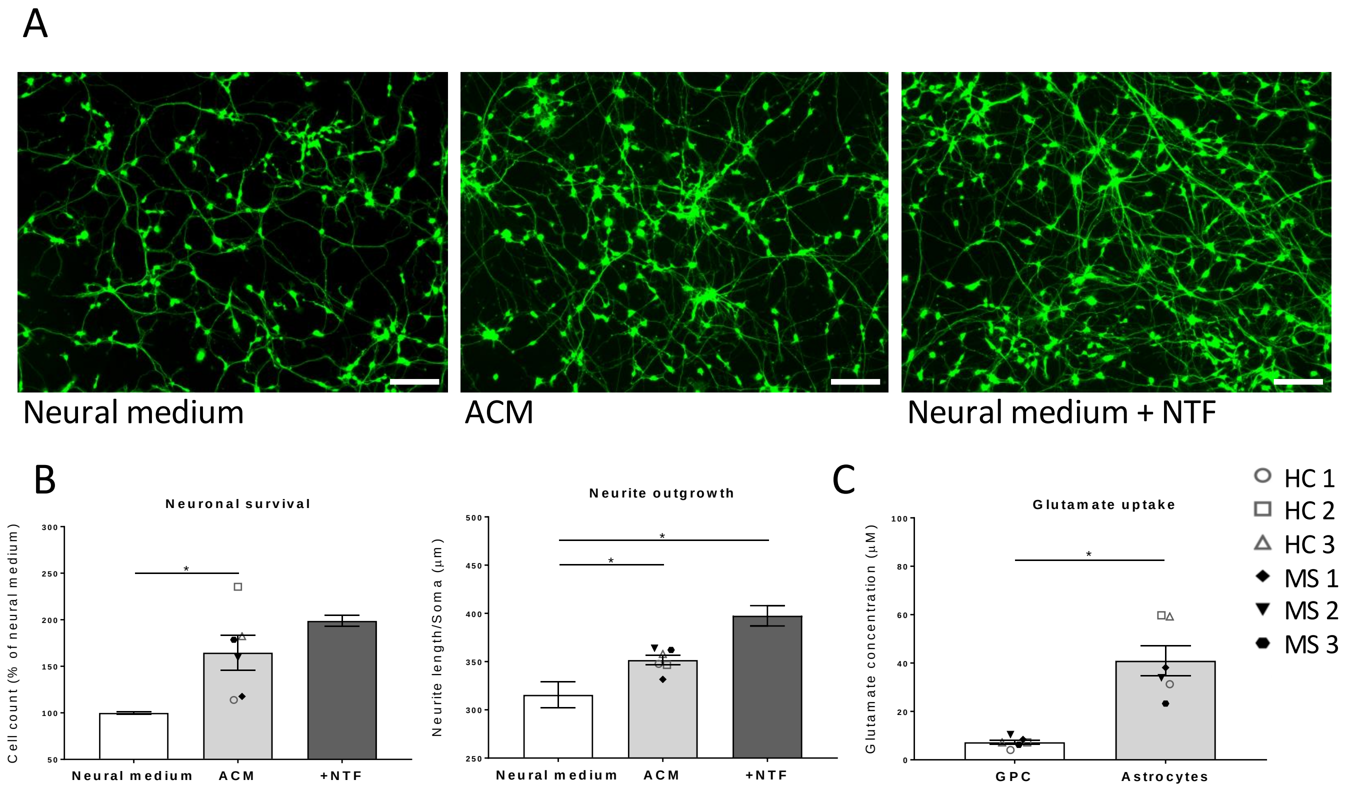


**Figure S4: Human iPSC-derived astrocytes support neuronal development and glutamate uptake**

(A) Representative pictures of neurons derived from healthy donor HC1 cultured either in standard culture condition (neural medium only), in astrocyte-conditioned neural medium (ACM) or in neural medium with addition of exogenous neurotrophic factors (+NTF, BDNF+GDNF), the optimal medium for neuronal development). Neurons were stained with calcein AM for fluorescence imaging. Scale bars: 200µm. Pictures were acquired with inverted microscope Axiovision Observer.Z1.
(B) Survival and neurite outgrowth of hiPSC-derived neurons were assessed after 7 days of culture in ACM as compared to standard culture condition (neural medium). For neurite outgrowth analysis, a minimum of 453 neurons per condition was analyzed.
(C) Capacity of mature astrocytes to take glutamate up was measured by enzymatic assay and compared for all the seven donors to matched glial precursors.
Each dot represents the mean results of one experiment performed in duplicate for each donor. Bars represent the global mean ± SEM. Wilcoxon test was performed to assess statistical significance ($p<0.05$: *).

## SUPPLEMENTAL TABLES

### Table S1: Media related to experimental procedures

| | Reagent | Final concentration | Supplier |
|---|---|---|---|
| **Erythroblast medium** | StemSpan SFEM medium | | StemCell technologies |
| | SCF | 50 ng/ml | RnD systems |
| | IGF-1 | 40 ng/ml | Miltenyi |
| | IL-3 | 10 ng/ml | Miltenyi |
| | Recombinant EPO | 2 U/ml | RnD systems |
| | Dexamethasone | 1 µM | Sigma-Aldrich |
| | Cholesterol Lipid Concentrate 250x (100mL) | 1x | Thermofisher |
| | Penicillin/Streptamycin | 1 :1000 | Bioconcept |
| **Neural induction medium** | DMEM/F-12 + Glutamax | | Gibco, Thermofisher |
| | N2 supplement | 1x | Gibco, Thermofisher |
| | B27 supplement without vitamin A | 1x | Gibco, Thermofisher |
| | Noggin | 500 ng/ml | Peprotech |
| | SB431542 | 20 µM | Tocris |
| | FGF-2 | 4 ng/ml | Peprotech |
| | Laminin | 2 µg/ml | Sigma-Aldrich |
| | Y-27632 | 10 µM | Miltenyi |
| **Neural expansion medium** | DMEM/F-12 + Glutamax | 50% | Gibco, Thermofisher |
| | Neurobasal medium | 50% | |
| | N2 supplement | 1x | Gibco, Thermofisher |
| | B27 supplement without vitamin A | 1x | Gibco, Thermofisher |
| | Laminin | 2 µg/ml | Sigma-Aldrich |
| | FGF-2 | 10 ng/ml | Peprotech |
| | EGF | 10 ng/ml | Miltenyi |
| | BDNF | 20 ng/ml | Peprotech |

**Table S1: Media related to experimental procedures (Continued)**

| | Reagent | Final concentration | Supplier |
|---|---|---|---|
| **Neural medium** | DMEM/F-12 + Glutamax | 50% | Gibco, Thermofisher |
| | Neurobasal medium | 50% | |
| | N2 supplement | 1x | Gibco, Thermofisher |
| | B27 supplement without vitamin A | 1x | Gibco, Thermofisher |
| | Laminin | 2 µg/ml | Sigma-Aldrich |
| **Glial expansion medium** | DMEM/F-12 + Glutamax | | Gibco, Thermofisher |
| | N2 supplement | 1x | Gibco, Thermofisher |
| | B27 supplement without vitamin A | 1x | Gibco, Thermofisher |
| | FGF-2 | 10 ng/ml | Peprotech |
| | EGF | 10 ng/ml | Miltenyi |
| | | | |
| **Astrocyte induction medium** | DMEM/F-12 + Glutamax | | Gibco, Thermofisher |
| | N2 supplement | 1x | Gibco, Thermofisher |
| | B27 supplement without vitamin A | 1x | Gibco, Thermofisher |
| | EGF | 10 ng/ml | Miltenyi |
| | LIF | 10 ng/ml | Peprotech |
| | | | |
| **Astrocyte medium** | DMEM/F-12 + Glutamax | | Gibco, Thermofisher |
| | B27 supplement without vitamin A | 1x | Gibco, Thermofisher |

**Table S2: Antibodies related to immunocytochemistry and flow cytometry**

| Antibody | Supplier | Reference |
|---|---|---|
| **Immunocytochemistry** | | |
| anti-Sox2 mouse IgG | Abcam | AB79351 |
| anti-Tra-1-60 mouse IgG | Millipore | MAB4360 |
| anti-Oct4 mouse IgG | Santa Cruz Biotechnology | sc-5279 |
| anti-SMA mouse IgG | Dako | M1110851 |
| anti-Pax6 rabbit IgG | Biolegend | PRB-278P |
| anti-AFP mouse IgG | Sigma-Aldrich | A8452 |
| anti-GFAP rabbit IgG | Sigma-Aldrich | AB5804 |
| anti-S100β rabbit IgG | Abcam | AB52642 |
| anti-GLAST rabbit IgG | Abcam | AB416 |
| anti-rabbit goat IgG AF488 | Thermofisher | A-21206 |
| anti-rabbit donkey IgG AF546 | Thermofisher | A10040 |
| anti-mouse donkey IgG AF546 | Thermofisher | A10036 |
| **Flow cytometry** | | |
| Anti-MHC-I FITC | Biolegend | sc-32235 |
| Anti-HLA-DR ECD | Beckman Coulter | IM3636 |
| Anti-GLAST APC | Miltenyi | 130-098-803 |
| Anti-GFAP Cy3 | Sigma-Aldrich | C9205 |
| Anti-S100β (rabbit) | Abcam | AB52642 |
| Anti-rabbit goat IgG AF488 | Thermofisher | A-21206 |

**Bead-based immunoassays**

**Affymetrix panel**: GM-CSF, TNFα, IL-1β, IL-1α, IL-12p70, IL-23p19, IFNβ, IFNα, IL-10

**RnD systems panel**: LIF, NRG-1β, HGF, PDGFα

## SUPPLEMENTAL EXPERIMENTAL PROCEDURES

### Reprogramming, culture and characterization of human iPSCs

For reprogramming into hiPSCs, 10 million PBMC were thawed and cultured for 4 days in erythroblast medium (see Supplementary material Table S1). CD71+ cells were sorted by MACS using anti-CD71 microbeads (Miltenyi) using the autoMACS system, then cultured for 7 days. CD71+ cells were nucleofected with three episomal plasmids (pCXLE-hOCT3/4-shp53-F, pCXLE-hSK and pCXLE-hUL, Addgene) (Okita et al. 2011) using the Amaxa Nucleofector (Lonza) and CD34 cell nucleofection kit (Lonza) and plated on Matrigel-coated plates (Corning) in the same medium. Medium was replenished every two days for a week with ReproTeSR medium (StemCell) and then changed every day for another week. Human iPSC clones started to appear from 14 days after nucleofection and onwards, and were individually picked and then cultured in TeSR-E8 medium (StemCell). Donor specific-iPSC lines were expanded in StemMACS iPSC-Brew XF medium (Miltenyi) and characterized by alkaline phosphatase assay and immunofluorescence imaging for pluripotency markers (see supplementary Table S2 for antibodies). Differentiation capacity was assessed by embryoid body formation in DMEM/F12+Glutamax medium (Gibco) supplemented with 20% FBS (Biowest) and stained for immunofluorescence imaging after 15 days.

### Immunocytochemistry

For immunocytochemistry, cells were plated on matrigel-coated 8-well slides (Merck-Millipore). Cells were washed once with PBS then fixed with 4% paraformaldehyde (PFA) diluted in PBS for 10 minutes at room temperature (RT) then washed 3 times with cold PBS. Fixed cells were blocked for 1 hour at RT in blocking buffer (PBS, normal goat serum 5%, saponin 0.2%) before overnight incubation at 4°C with primary antibodies diluted in blocking buffer. The next day, cells were washed 3 times with PBS and incubated for 1 hour at RT with secondary antibodies. After 3 washes in PBS, slides were incubated with DAPI (1:500 in PBS) for 10 minutes then washed once with PBS. All antibodies used are detailed in the supplementary material, Table S2. Finally, microscope slides were mounted with Diamond anti-fade Prolong medium (Life technologies). Images were acquired on a confocal Zeiss LSM 880 microscope or inverted microscope Axiovision Observer.Z1 and analyzed with Zen 2.1 software.

### Flow cytometry

For flow cytometry staining, astrocytes grown in Astrocyte Medium were harvested with trypsin, then stained as previously described (Perriard et al. 2015). Briefly, cells were first stained with violet Live/Dead (Life technologies) for 20 minutes at 4°C. For extracellular staining, cells were resuspended in PBS-2% FBS and stained with antibodies for 30 minutes at 4°C. For intracellular staining, cells were incubated for 20 minutes at 4°C in Cytofix/Cytoperm buffer (BD Biosciences) then washed with permwash buffer 1x (BD Biosciences) and stained for 20 minutes at 4°C with primary antibodies. Cells were then washed and stained with secondary antibodies

for 20 minutes at 4°C. All antibodies used are detailed in the supplementary material, Table S2. Data were acquired on a LSRII flow cytometer (BD Biosciences) and analyzed with FlowJo software (version 9.1.11, Treestar).

**Calcium transients**

Astrocytes were washed once with HBSS then loaded with Fluo-4AM (2µM, Life Technnologies) for 15min at 37°C. Astrocytes were then washed again with culture medium and imaged immediately. Live imaging was performed with inverted microscope Axiovision Observer.Z1. Cells were imaged for 120s after ATP (3µM) addition and one picture was taken every 683ms. Data were analyzed with Fiji (ImageJ 2.0.0). Cells were individually outlined as region of interest (ROI) and fluorescence intensity quantified over time. For each ROI, data were normalized by the lowest value of fluorescence and responding cells were quantified as having at least one calcium transient. A minimum of 56 ROIs were analyzed per donor/cell line.

**Neurite outgrowth assay**

To study the impact of components secreted by astrocytes on neurite outgrowth, astrocytes generated from each of the donor-specific iPSC lines were cultured in neural medium for 48 hours. Culture supernatants were harvested and stored at -20°C until use. As astrocytes may have consumed essential nutriments, astrocyte-conditioned supernatants were replenished with fresh neural medium and concentrated 10 times using Amicon 30kDa columns (Millipore). These ACM were finally used for neurite outgrowth assays described hereafter. Neurons were differentiated from the hiPSC line C14 of the healthy control HC 1. After 10 days of differentiation, neurons were passaged with Accuse and seeded at 50’000 cells/cm² in neural medium supplemented with Laminin (2µg/ml) and Y-27632 (10µM) on PO/L-coated plates. Cells were pretreated with Z-VAD-FMK (1µM, Sigma Aldrich) for 30 minutes prior to passage to increase cell survival. The next day, medium was changed to ACM, neural medium only (negative control) or neural medium + BDNF (20ng/ml) + GDNF (10ng/ml) (positive control). After five days of culture, neurons were washed with PBS then stained with Calcein AM (2µM, Life Technnologies) for 20 minutes prior to live imaging. To assess neuronal survival, neurons were harvested with trypsin and counted. Results were normalized with control condition in neural medium only. Pictures were analyzed with the ImageJ plugin NeurphologyJ (Ho et al., 2011) for neurite measurement.

**Glutamate assay**

For glutamate uptake, cells were washed once with HBSS buffer (Ca2+, Mg2+, Gibco) and left to equilibrate for 10 minutes. After a second wash, they were incubated in HBSS buffer supplemented with L-Glutamate (100µM, Sigma-Aldrich) for 1 hour at 37°C. Supernatants were then harvested and centrifuged for 10 minutes at 2000g. Glutamate concentration was measured with a glutamate assay kit (Sigma Aldrich). Absorbance was measured with Opsys MR microplate reader from Dynex Technologies.
